# Supplementary material for: The interactome of intact mitochondria by cross-linking mass spectrometry provides evidence for coexisting respiratory supercomplexes
Source: Mol Cell Proteomics. 2017 Dec 8;17(2):216–32. doi: 10.1074/mcp.RA117.000470 (PMC5795388; doi:10.1074/mcp.RA117.000470)
Supplement: Supplemental Data [file supp_RA117.000470_133922_0_supp_23978_fzffwf.zip › spectra_annotation/mito_spectra_annotation/52-1-8-1-9-1.pdf]

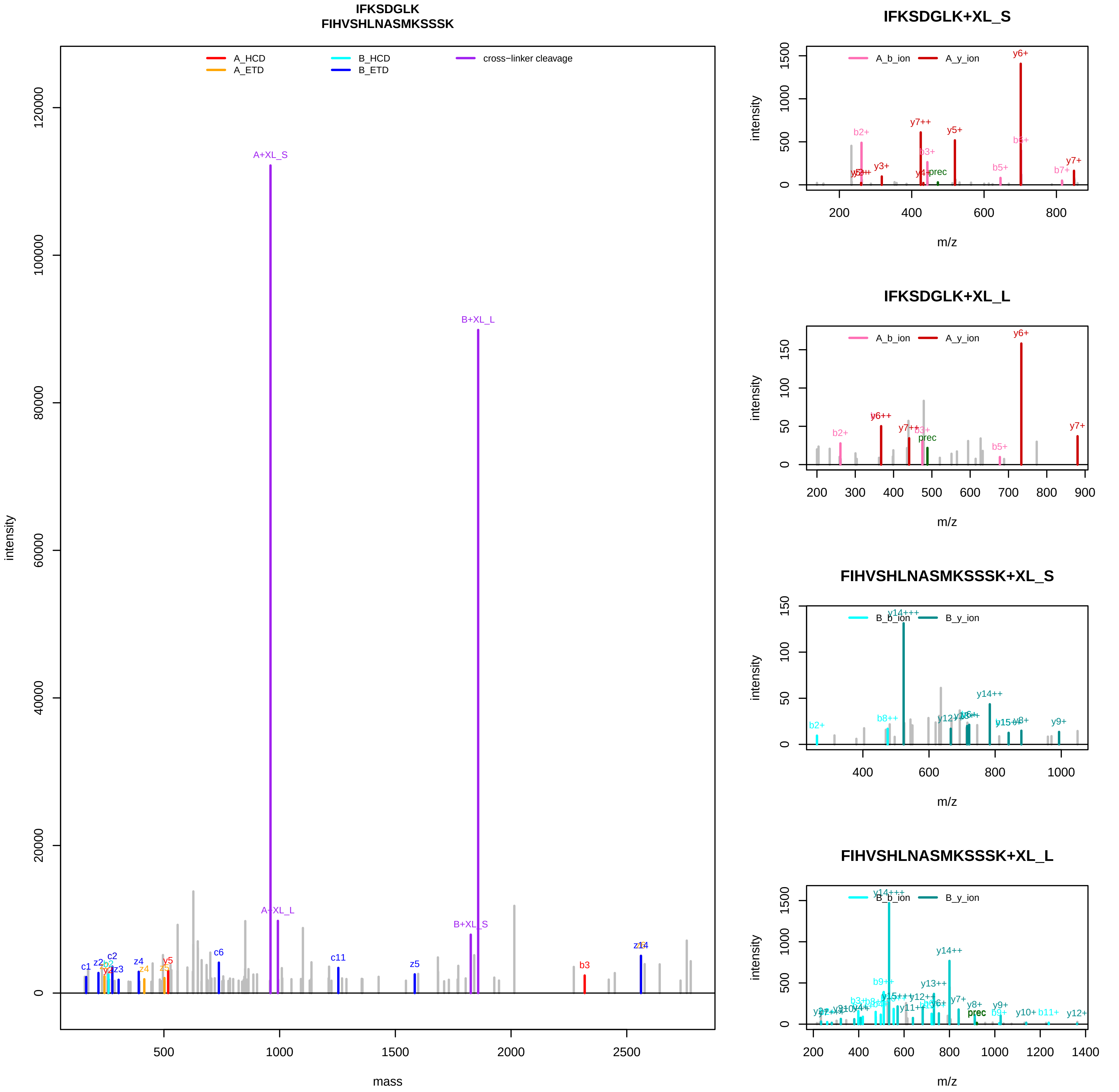
IFKSDGLK
FIHVSHLNASMKSSSK
A_HCD
A_ETD
B_HCD
B_ETD
cross−linker cleavage
intensity
mass
A+XL_S
B+XL_L
A+XL_L
B+XL_S
c1
z2
c2
z3
z4
z4
y5
c6
c11
z5
b3
z14
IFKSDGLK+XL_S
A_b_ion
A_y_ion
y6+
y7++
y5+
b2+
y3+
b3+
prec
b5+
b7+
y7+
m/z
IFKSDGLK+XL_L
y6++
y7++
b2+
b5+
FIHVSHLNASMKSSSK+XL_S
B_b_ion
B_y_ion
y14+++
y14++
b8++
b2+
y9+
FIHVSHLNASMKSSSK+XL_L
y13++
b9++
y7+
y8+
y9+
y10+
b11+
y12+
